# Molecular Markers of Pancreatic Cancer: A 10-Year Retrospective Review of Molecular Advances

**DOI:** 10.7759/cureus.29485

**Published:** 2022-09-23

**Authors:** Boma E Jacks, Chinwendum U Ekpemiro, Adeyemi A Adeosun, Uchechukwu O Ogbonna, Faithful T Ogundiran, Funmilola Babalola, Nkemputaife P Onyechi, Olamide O Ajayi, Maureen G Boms, Adaugo N Nwanguma, Uduak A Udo, Okelue E Okobi, Evidence E Ohikhuai, Endurance O Evbayekha

**Affiliations:** 1 Surgery, University of Maiduguri Teaching Hospital, Maiduguri, NGA; 2 Surgery, Federal Medical Centre, Umuahia, NGA; 3 Molecular Pharmacology and Experimental Therapeutics, Mayo Clinic, Rochester, USA; 4 Surgery, Barau Dikko Teaching Hospital, Kaduna, NGA; 5 Surgery, All Saints University School of Medicine, Roseau, DMA; 6 Epidemiology and Public Health, Texas Department of State Health Services, San Antonio, USA; 7 Internal Medicine, University Hospitals Cleveland Medical Center, Cleveland, USA; 8 Internal Medicine, Obafemi Awolowo College of Health Sciences, Olabisi Onabanjo University, Sagamu, NGA; 9 Clinical Research, University of Alabama at Birmingham, Birmingham, USA; 10 Medicine and Surgery, Nnamdi Azikiwe University College of Health Sciences, Nnewi, NGA; 11 Public Health, Washington University in St. Louis, St. Louis, USA; 12 Radiology, Imaging for Women, Kansas City, USA; 13 Family Medicine, Lakeside Medical Center, Belle Glade, USA; 14 Public Health, Jackson State University School of Public Health, Mississippi, USA; 15 Internal Medicine, St. Luke's Hospital, St. Louis, USA

**Keywords:** molecular advances, epigenetics, pancreatic cancer, biomarkers multiomics, tumor markers

## Abstract

Pancreatic cancer remains the third leading cause of death amongst men and women in the United States. Pancreatic ductal adenocarcinoma (PDAC), the most common type of pancreatic cancer maintains its reputation of being the most aggressive with a poor prognosis. One of the contributing factors to the high mortality of PDAC is the absence of biomarkers for early detection of disease and the complexity of tumor biology and genomics. In this review, we explored the current understanding of epigenetics and diagnostic biomarkers in PDAC and summarized recent advances in molecular biology. We discussed current guidelines on diagnosis, prognosis, and treatment, especially in high-risk individuals. We also reviewed studies that have touched on identifying biomarkers and the role they play in making early diagnosis although there are currently no screening tools for PDAC. We explored the recent understanding of epigenetic alterations of PDAC and the future implications for early detection and prognosis. In conclusion, the new and emerging advances in the detection and treatment of PDAC can lead to an improvement in the current outcome of PDAC.

## Introduction and background

Despite years of research and advances in understanding the disease mechanisms, Pancreatic Ductal Adenocarcinoma (PDAC) maintains its reputation as an aggressive disease with a poor prognosis, accounting for almost as many deaths as new cases yearly, according to the Global Cancer Incidence, Mortality and Prevalence (GLOBOCAN) statistics [1]*.* The incidence of PDAC increases steadily by decade after age 40, classically being diagnosed in the 7th decade, with only about 10% of cases in individuals under 55 years [2,3]*. *Pancreatic cancer is the third leading cause of death in men and women. The worldwide five-year survival for pancreatic cancer ranges from 2-9% [4-6].

The United States has a five-year relative survival rate of 11%, the lowest amongst solid tumor malignancies [7]. This contrasts with Pancreatic Neuroendocrine Tumors (PNET), which mostly run an indolent course [8]. However, it can be argued that PDAC initially runs an indolent course as well, as PDAC is largely asymptomatic before advanced disease, contributing to its high mortality rate [9]*.* Other factors contributing to the high mortality of PDAC include the absence of biomarkers for early disease detection and the complexity of tumor biology and genomics, limiting the effectiveness of conventional medical therapy [9,10].* *There are also complexities in the genetic mutations associated with PDAC; for instance, the pattern of the major genetic mutation seen in PDAC, that is, the activation of the oncogene KRAS is distinct from other malignancies in that KRAS mutation is an inciting event in PDAC, and is seen in the precursor lesions, Pancreatic intraepithelial neoplasia (PanIN). In contrast, it emerges as a late oncogenic driver after epigenetic changes have been created by founder mutations in other malignancies [3]. Other genetic mutations driving PDAC are the inactivation of the tumor suppressors *TP53* and *CDKN2A*, and attempts at targeting therapy against these mutations have proved futile [9]. 

Considering these, it goes without saying that to improve the prognosis of PDAC, we need early diagnostic biomarkers and other modalities of treatment that target epigenetic alterations. Therefore, this review explores the current understanding of epigenetics and diagnostic biomarkers in PDAC. It also summarizes recent advances in molecular biology and their therapeutic and prognostic implications in pancreatic cancer.

## Review

Methodology

We employed a preset standard for our data search, selection, and inclusion. The search was from the repositories of PubMed, Google Scholar, and ScienceDirect Library. Articles included were from the last ten years, i.e., 2012-2022. They included randomized clinical trials, meta-analyses, systematic reviews, and other observational studies. 

Our keywords for the search were: (tumor markers) (biomarkers multiomics) (pancreatic cancer). We used the search engine layout to generate all possible pieces of literature with these word combinations. The google scholar search initially returned a total of 6,830 results; To trim down to the most specific articles, we employed the ‘advance search’ on google scholar. The advanced search using ‘tumor marker’ for ‘exact word’ resulted in 51 articles, ‘biomarkers in pancreatic cancer’ returned 44 article search results, and ‘multi-omics in pancreatic’ returned three article results. Using the same ‘advanced search’ approach, we searched PubMed and ScienceDirect and found 32 and 17 related articles, respectively.

Eligibility Criteria

**Figure 1:**
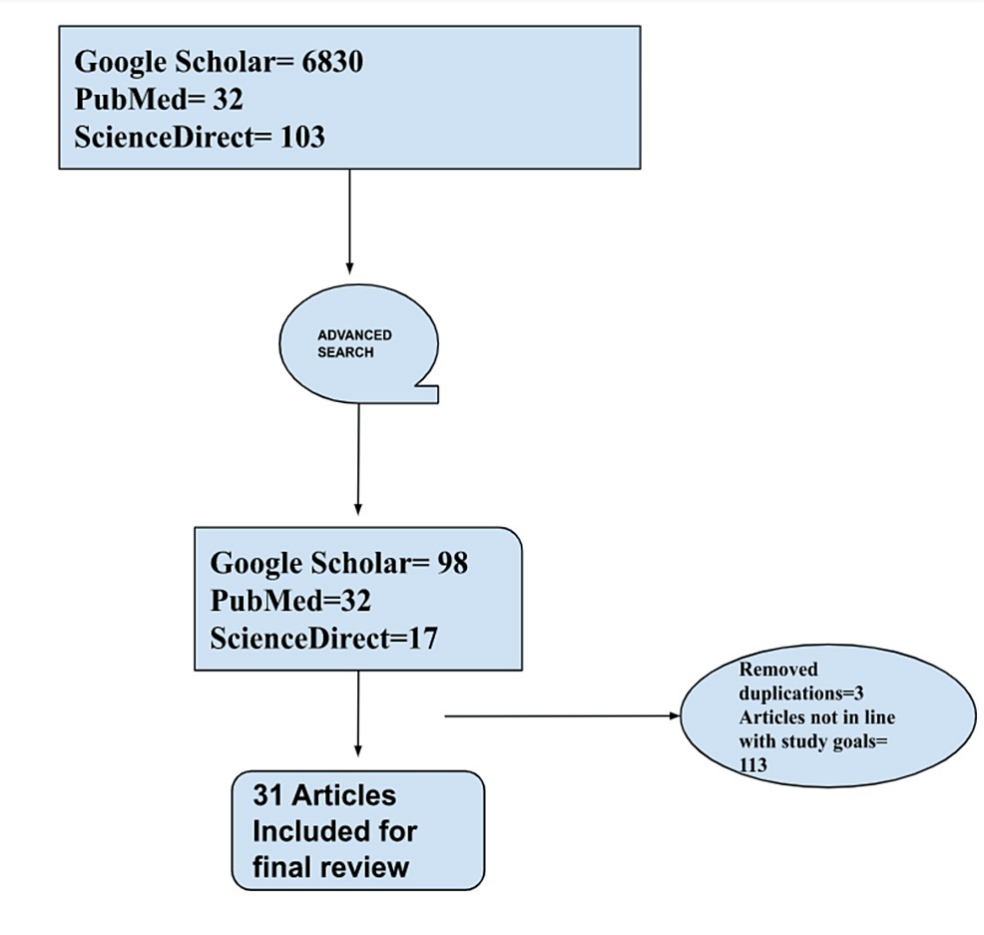
Flowchart illustration for literature selection and review

A total of 147 articles were screened by five independent contributors, first using the contents of the abstract and then a full-text read. A conflict of inclusion or exclusion between the contributors was settled by the vote of the fifth contributor. We further narrowed the articles to 31, and we analyzed the studies according to the various facets of the interest of our study. A concise inclusion and exclusion criteria are shown in Table 1, and a flowchart for article screening is demonstrated in Figure 1.

**Table 1:** Eligibility Criteria For Our Literature Search

Inclusion criteria	Exclusion criteria
1) Literature relevant to epigenetics of pancreatic cancer	1) The studies that discussed the pancreas but did not discuss all or some parts of its epigenetics were excluded because the objective of the study was focused on the epigenetics of pancreatic cancer
2) The studies must be original (randomized clinical trials, systematic reviews, meta-analyses) in which the epigenetics of pancreatic cancer were identified or discussed.	2) Opinion pieces and non-scholarly articles, secondary studies, scoping reviews, and research approaches other than primary studies were excluded.
3) Human studies	3) Animal studies
4) The studies must be published in a peer-reviewed journal to maintain the validity and reliability of the studies.	4) The studies that were published in non-peer-reviewed journals, and dissertations, were excluded.
5) The studies must be originally published in English for readability by the reviewers.	5) The studies originally published in a language other than English were discarded.
6) Works of literature published within the last ten years (2012–2022).	

Genetics and epigenetics in pancreatic cancer

Various studies have been carried out to identify genes that play a role in pancreatic cancer. A study was carried out by Waddell et al., in which the genome sequencing of 100 pancreatic ductal adenocarcinomas was done [11]. They concluded that variation in chromosomal structure is an important mechanism of DNA damage in pancreatic carcinogenesis. They identified commonly mutated genes that characterize pancreatic ductal adenocarcinomas (*KRAS*, *TP53*, *SMAD4*, and *CDKN2A*) and also identified two additional genes not previously described in human pancreatic ductal adenocarcinomas (*KDM6A* and *PREX*) [11-13]. Another study was performed by Li et al. to identify hub genes that play a role in pancreatic cancer diagnosis and management [14]. They integrated two microarray data sets from the GEO database and identified 11 genes that were significantly different in tumor samples compared with normal samples which were screened. They identified 2 Hub genes Matrix Metallopeptidase (*MMPS*) 7 and integrin, alpha 2 which are significantly different in normal tissues versus tumor samples and can be used in diagnosis and therapy in the future. They stated that *MMPS* is involved in cell proliferation, migration, and differentiation and may correlate with cancer activity and poor prognosis. The interaction between them and extracellular ligands induced a signaling cascade that regulated intracellular and extracellular activities [14-16].

Epigenetics refers to the changes in the phenotype which affect gene expression without altering the DNA sequence. A study by Khoshchehreh et al. emphasizes the role of epigenetics reprogramming in the tumorigenicity of pancreatic adenocarcinoma [17]. Their rationale for the study was that although cancer cells develop through genetic mutations, the genes acquire epigenetic abnormalities that affect their expression. They tested three somatic cell reprogramming methods on their pancreatic ductal adenoma primary cancer cultures that do not integrate into the genome; they found that reprogramming the epigenome significantly affected the tumorigenicity of PDAC and its differentiation status and expression [17-19].

Epigenetic changes are reversible, making them targets for tumor-directed therapies. They occur through the methylation of DNA, histone modifications, and changes in chromatin structures. Epigenetic regulation and the corresponding changed chromatin states frequently occur in genes partaking in oncogenic signaling, metabolic alterations, and the metastatic process. [9,20-26]. These changes influence tumor development and progression by activating oncogenes and shutting down tumor suppressor genes. For instance, adipokines' paracrine activation of the non-canonical Wnt signaling pathway receptor tyrosine kinase-like orphan receptor 2 (ROR2) leads to early epithelial to mesenchymal transition (EMT) and has been postulated to be a possible mechanism for early metastasis. This activation is modulated by various non-coding RNAs and chromatin modifiers under epigenetic control. Also, epigenetic alterations occur early in carcinogenesis. The DNA methylome in PDAC is significantly altered from healthy controls, with several protein-coding genes and long non-coding genes being potential diagnostic biomarkers for subclinical disease [9,23,24,27].

Epigenetic modifications are also responsible for the heterogeneous tumor microenvironment in PDAC. The changes drive tumor desmoplasia and induce the creation of a hypoxic and immunosuppressive tumor microenvironment (TME) with a lack of T-cell infiltration and resistance to immune-checkpoint inhibition (ICI) therapy. These immune modifications are thought to be a result of transcriptional regulations driven by the activation of ubiquitin-specific protease 22 (*USP22*) and stromal desmoplasia driven by focal adhesion kinase (*FAK*). Increased lactate levels and suppression of lysine demethylase 3A (*KDM3A*) are thought to be the mechanism for decreased T-cell infiltration [3,9,23,24]. Epigenetic changes may also be specific to particular molecular subtypes of PDAC and provide the opportunity for therapeutic intervention [9,23,27].

Pancreatic cancer diagnosis, treatment, prognosis, and the need for biomarkers

Pancreatic cancer is diagnosed based on patient history, Imaging, and laboratory testing. Commonly used imaging modalities include contrast-enhanced abdominal CT, transabdominal and endoscopic ultrasound, Endoscopic Retrograde Cholangiopancreatography (ERCP), and MRI [9,23]. Surgical resection is the only curative option in PDAC, however, only about 20% of patients present with the surgically amenable disease. For patients with advanced disease, adjuvant therapy with FOLFIRINOX (5-fluorouracil, irinotecan, leucovorin, oxaliplatin) or gemcitabine plus albumin-bound paclitaxel combination has been found to increase median survival from 6.8 months with gemcitabine alone to 11.1 months 8.5 months and with FOLFORINOX and gemcitabine and nab-paclitaxel combination respectively. Despite this, the five-year survival for patients with nonresectable pancreatic cancer is approximately 9% [7,25]. Due to the asymptomatic course of pancreatic cancer and its poor prognosis, the role of screening in high-risk patients is paramount in improving disease-free survival. 

In its updated guidelines, the International Cancer of the Pancreas Screening Consortium came to a consensus that screening for pancreatic cancer in patients with a familial risk should begin no earlier than 50 or 10 years earlier than the youngest relative with pancreatic cancer. MRI/magnetic retrograde cholangiopancreatography (MRCP) and/or endoscopic ultrasound (EUS) were the preferred diagnostic modalities [27,28]. The benefits of early detection were shown in a 16-year study on high-risk individuals for pancreatic cancer, where nine of 10 tumors detected by surveillance were resectable with improved short-term patient outcomes [15,23,27]. However, only about 5-10% of patients with pancreatic cancer are genetically predisposed to developing the disease [3,28]. This need for early detection of PDAC has driven the search for clinically relevant diagnostic and prognostic biomarkers. Currently, the sole FDA-approved biomarker for PDAC is serum Cancer Antigen (CA)19-9, mostly used for disease monitoring rather than screening due to inherent limits of sensitivity and specificity: CA19-9 levels can be elevated in several conditions unrelated to pancreatic cancer, while subjects lacking the Lewis-A antigen do not produce CA19-9 at all [6,21].

Future trends in diagnosis and prognostication of pancreatic adenocarcinoma

A more recent understanding of epigenetic alterations and the heterogenous tumor microenvironment of pancreatic adenocarcinoma have paved the way for promising biomarkers with potential for diagnosis and predicting both survival and response to therapeutic interventions. These include selectively expressed DNA methylation biomarkers, circulating and tissue-bound transcriptomes, and fecal microbiota signatures. Eissa et al. used circulating cell-free DNA (cfDNA) from a large cohort of patients at different stages of pancreatic cancer and an age-matched normal group to study DNA methylation of genes *ADAMTS1* and *BNC1* to determine the usefulness of this two-gene panel as a non-invasive biomarker set for the early detection of pancreatic cancer. The two-gene panel (*ADAMTS1 *and/or *BNC1*) was positive in 100% (8/8) of stage I, 88.9% (8/9) of stage IIA, and 100% (20/20) of stage IIB disease. The sensitivity and specificity of the two-gene panel for localized pancreatic cancer (stages I and II), where the cancer is eligible for surgical resection with curative potential, was 94.8% and 91.6%, respectively [17,21,28]. Thus, indicating that DNA methylation-based biomarkers may be valuable as non-invasive tests for early detection of PDAC [28].

Circulating transcriptome contains diverse non-coding, stable and functional elements such as microRNAs (miRNAs), long non-coding RNAs (lncRNAs), and circular RNAs (circRNAs). Their resistance to RNase activity and readiness to be detected in the biological fluids of cancer patients showcase their potential as biomarkers in PDAC. Profiling miRNA expression can correlate to the stage of malignant pancreatic disease and hold potential as diagnostic and prognostic markers [9,23,27]. Various RNA types, including messenger RNA (mRNA), circRNA, and lncRNA, can be found in blood and urine extracellular vesicles/exosomes (EV). Collectively termed long RNA (exLRs), EV-associated exLRs exhibit different profiles in patients with PDAC and healthy controls and are a potential diagnostic tool. They may detect PDAC at an early resectable stage and, importantly, can detect PDAC in patients without elevated levels of the tumor marker CA19-9 [9,23,29].

An increased ratio of mIR-3940-5p/miR-8069 detected in urine exosomes has been reported in the early stages of PDAC. With improved diagnostic accuracy when combined with CA19-9 (sensitivity of 93.0% and positive predictive value {PPV} 78.4%, increasing to a PPV of 100% when all markers were positive) [9,23,27]. Some long non-coding RNAs (lncRNAs) like the Hox transcript antisense RNA (HOTAIR) lncRNA and hexokinase-2 (HK2) expression levels are elevated in the serum of patients with PDAC compared to healthy controls, and their detection in plasma or serum have been explored as potential diagnostic biomarkers [9,23,27].

Also, high expression levels of HOTAIR and HK2 have been associated with significantly lower overall survival rates than patients with low expression levels. Conversely, low expression levels of lncRNA LINC01111, which acts as a tumor suppressor, are associated with a poor prognosis [9,23,27]. Mishra et al. analyzed multi-omics data from the cancer genome atlas and also found that high expression of the lncRNA LINC00941correlated with poor prognosis [20]. Owing to their abundance, conservation, stability within exosomes, and specificity in tissues and cells, circRNAs have shown promise as potential prognostic biomarkers for pancreatic cancer. High expressions of circ_0007534, circ-ADAM9, circ-ASH2L, circ-LDLRAD3, circ -PDE8A (hsa_circ_0036627) have each been independently associated with worse prognosis in PDAC [29]. Yang et al. also confirmed that circ-LDLRAD3 was significantly upregulated in pancreatic cancer tissues and plasma and that a high level of circ-LDLRAD3 was positively associated with tumor venous invasion (p=0.025) and lymphatic metastasis (p=0.014). Its expression in plasma was significantly associated with CA19-9 levels (p=0.03), N stage (p=0.049), venous invasion (p=0.005), and lymphatic metastasis (p=0.014) [20,29].

Research into the tumor microenvironment and metabolic pathways associated with the progression of pancreatic cancer has opened up a new direction for diagnosis and prognosis in patients with pancreatic adenocarcinoma [30]. Li et al. (2021) investigated the expression and prognostic value of the S100A family of calcium-binding proteins in four PDAC cell lines and tissues of PDAC patients undergoing surgery using real-time polymerase chain reaction (PCR). They noted that S100A2, S100A4, S100A6, S100A10, S100A14, and S100A16 were over-expressed in PDAC compared to normal samples. They also observed that higher mRNA expression of S100A2/10/14/16 was significantly associated with shorter overall survival OS in PDAC patients (P< 0.05) [21,30]. Guangwei et al. performed a bioinformatic analysis of the cancer genome atlas (TCGA) to identify glycolysis-related genes involved in PDAC and identified 13 independent prognostic genes, including *MET*, *B3GNT3*, *SPAG4*, *RPE*, *KIF20A*, *CDK1*, *PGK1*, *AURKA*, *P4HA1*, *PYGB*, *HMMR*, *SDC1*, and *EFNA3*. Based on these results, they developed a three-gene signature (*MET*, *B3GNT3*, and *SPAG4*) and risk score for prognosis prediction of PDAC. Patients were divided into high-risk and low-risk groups based on their scores. The overall survival (OS) was significantly poorer in the high-risk group than in the low-risk group (p < 0.000) The area under the curve (AUC) for one-year OS was 0.764. They also found that when combined with other variables like age, gender, tumor stage, radiotherapy, and tumor residual, the scores obtained predicted survival time (C-index 0.67 {SE}=0.031) in a way that showed potential suitability for clinical application [12,27].

Karasinka et al., in another study, investigated metabolic variants of PDAC using RNA data from resectable and nonresectable tumors to determine the relative expression of the cholesterol and glycolytic pathway genes in a quest to determine their relationship with OS. They stratified them into four metabolic subgroups: quiescent, glycolytic, cholesterogenic, and mixed. They also noted significant differences in survival and metastatic potential between the subgroup. The glycolytic profile was associated with the shortest median survival in both resectable (log-rank test p=0.018) and metastatic (log-rank test p=0.027). Interestingly, a survival benefit was observed in cases with increased expression of cholesterol synthesis genes. Cholesterogenic cases had the longest median overall survival in the resectable (log-rank test p=0.0031 vs. glycolytic, p=0.043 vs. mixed, p=0.025 vs. quiescent) and metastatic (log-rank test p=0.011 vs. glycolytic) groups. Cholesterogenic cases had the longest relapse interval in resected PDAC [31-33]. Thus, metabolic gene profiles may have benefits as markers of diagnosis, tumor aggressiveness, and prognosis in PDAC. Some researchers have also explored differences in microbiomes to identify potential markers for early diagnosis of PDAC. Kartal et al. studied fecal microbiota and identified PDAC with an accuracy of 0.84 area under the receiver operating characteristic curve (AUROC) in a Spanish cohort based on 27 species [25]. The accuracy improved to 0.94 AUROC when combined with the less specific carbohydrate antigen CA19-9 serum marker. The classifier was validated in an independent German PDAC cohort (0.83 AUROC), and PDAC disease specificity was confirmed against 25 publicly available metagenomic study populations with various health conditions (n=5792). Marker taxa enriched in fecal samples (Veillonella, Streptococcus, Akkermansia) and taxa with differential abundance in healthy and tumour pancreatic tissues (Bacteroides, Lactobacillus, Bifidobacterium) were validated by fluorescence in situ hybridization. The described fecal microbiome signatures enabled robust metagenomic classifiers for PDAC detection at high disease specificity, complementary to existing markers, and potential for cost-effective PDAC screening and monitoring [32-34].

## Conclusions

This review highlights recent advances in molecular biology and the therapeutic and prognostic implications in pancreatic cancer. With advances in technology over the last 10 years, there have been new biomarker discoveries geared toward improving the outcome of patients with PDAC. We discovered that the recent understanding of the epigenetics of PDAC had paved the way for promising biomarkers with diagnostic and prognostic value. Researchers have found that DNA methylation-based biomarkers, transcriptomes, metabolic gene profiles, and fecal microbiomes are valuable in the early detection, diagnosis, and prognosis of PDAC. We also found that some newly discovered biomarkers can detect tumor aggressiveness and predict overall survival. These new advances in early detection should inform scientific research geared towards improving overall outcomes in PDAC patients.
